# Efficacy and Safety of Re Du Ning Injection for Acute Exacerbations of Chronic Obstructive Pulmonary Disease: A Systematic Review and Meta-Analysis

**DOI:** 10.1155/2022/7479639

**Published:** 2022-03-21

**Authors:** Bofei Shu, Hong Li, Xu Zhou, Zhaohui Ding, Liling Wan

**Affiliations:** ^1^Graduate School, Jiangxi University of Chinese Medicine, Nanchang, Jiangxi, China; ^2^Evidence-based Medicine Research Centre, Jiangxi University of Chinese Medicine, Nanchang, Jiangxi, China; ^3^Department of Respiratory Medicine, The Affiliated Hospital of Jiangxi University of Chinese Medicine, Nanchang, Jiangxi, China

## Abstract

**Background:**

Re Du Ning, a traditional Chinese medicine injection, has been widely used for the treatment of chronic obstructive pulmonary disease, although without established systematic review evidence. This systematic review aimed to assess the efficacy and safety of Re Du Ning in the treatment of acute exacerbations of chronic obstructive pulmonary disease (AECOPD).

**Methods:**

We searched seven databases (PubMed, Embase, the Cochrane Library, SinoMed, CNKI, WanFang, and the Chinese Clinical Trial Registry) up to November 1, 2021, to identify randomized controlled trials of Re Du Ning for AECOPD. Two researchers independently carried out literature screening and data extraction. Effects were measured by risk ratios (RRs) or mean differences (MDs) with 95% confidence intervals (CIs). The meta-analysis was completed by RevMan 5.4 software.

**Results:**

Twenty-six studies met the eligibility criteria, with a total of 2284 patients. The findings of the meta-analysis indicated that the response rate of the experimental group was higher than that of the control group: RR = 1.14% and 95% CI: (1.09, 1.19). Significantly greater improvements in pulmonary function: FEV1: MD = 0.28 L, 95% CI: (0.20, 0.36); FEV1/FVC: MD = 8.63%, 95% CI: (4.68, 12.59); T-lymphocyte counts: CD4: MD = 6%, 95% CI: (2.44, 9.56); CD3: MD = 10.42%, 95% CI: (8.6, 12.24); CD4/CD8: MD = 0.38%, 95% CI: (0.32, 0.43); acid/base imbalance: PH: MD = 0.05, 95% CI: (0.01, 0.10); PaO2: MD = 9.02 mmHg, 95% CI: (11.11, 0.10), *p*=0.005; C-reactive protein: MD = −6.65 mg/L, 95% CI: (−10.97, −2.34); and PCT: MD = −0.28 *μ*g/L, 95% (CI: −0.41, −0.15) were observed in patients receiving Re Du Ning compared with those receiving the control treatment. Re Du Ning did not significantly change the carbon dioxide partial pressure. All reported adverse reactions were mild.

**Conclusion:**

Re Du Ning injection, as a complementary therapy to routine treatment, has better efficacy than Western medicine alone in relieving clinical symptoms, delaying pulmonary function decline, and improving inflammation indicators for AECOPD, with good safety. The evidence was limited by a lack of high-quality RCTs.

## 1. Introduction

Chronic obstructive pulmonary disease (COPD) is a common disease characterized by continuous airflow limitation. COPD is related to the increased chronic inflammatory response of the respiratory tract and lungs to toxic particles or gases. According to the 2021 Global Initiative for Chronic Obstructive Lung Disease, COPD has become one of the top causes of death in the world [1]. An 8-year retrospective cohort study showed that the economic losses caused by COPD amount to 50 billion dollars per year in Europe [2]. The 2019 Asia-Pacific Respiratory Association's position statement on COPD management in Asia pointed out the shortcomings of COPD prevention and treatment in underdeveloped regions, namely, insufficient awareness, insufficient diagnosis, and insufficient treatment [3]. Therefore, the control of COPD in developing countries is even more suboptimal.

Acute exacerbations of COPD (AECOPD) lead to significant damage to pulmonary function, with a risk of readmission after discharge as high as 58% and mortality within one year of up to 20–25% [4]. Patients with COPD experience an average of one to four acute exacerbations per year. How to control symptoms, improve the quality of life, and reduce costs are crucial issues in the management of AECOPD. However, the American Thoracic Association's 2020 clinical guidelines pointed out that there was no evidence on the efficacy of the routine multidrug regimen (bronchodilators + hormones + antibiotics) for deterioration of COPD [5]. The long-term use of hormones also significantly increases the risk of secondary pneumonia, oral *Candida* infection, osteoporosis, and diabetes [6, 7]. Therefore, the European Respiratory Association proposed reducing the use of steroids for COPD and hopefully finding alternative therapies to improve the efficacy and reduce the incidence of adverse reactions [8].

Traditional Chinese medicine is increasingly attracting attention in the field of adjuvant therapy for respiratory diseases. As one of the injectable products, Re Du Ning has been listed in twelve guidelines of respiratory diseases such as H1N1 influenza, children viral pneumonia, and Middle East respiratory syndrome [9]. The injection is extracted from *Artemisia annua*, honeysuckle, and *Gardenia*, and these herbs have the effects of clearing away heat, dispelling wind, and detoxifying based on the theory of traditional Chinese medicine. Re Du Ning has been clinically used for alleviating symptoms caused by upper respiratory tract infections, such as high fever, aversion to cold, head and body pain, cough, and yellow sputum. In an animal experiment, Re Du Ning showed antipyretic, anti-inflammatory, and antiviral effects [10]. The efficacy of Re Du Ning injection for community-acquired pneumonia and bronchial asthma in the human body has also been proven [11]. However, there is no consensus on the use of Re Du Ning in the treatment of COPD.

We thus performed this systematic review to include and incorporate all currently available RCTs regarding Re Du Ning injection for AECOPD by meta-analytic techniques to provide evidence with rigorous assessments for clinical application. The study protocol was submitted to the PROSPERO registration platform (number: CRD42021279153).

## 2. Methods

### 2.1. Inclusion and Exclusion Criteria

The study population was set as adults who were diagnosed with COPD by any recognized international guidelines and developed acute exacerbations. The intervention in the experimental group was Re Du Ning combined with Western medicine, and the control group was treated with Western medicine alone. Eligible Western medicine included antibiotics, phlegm-reducing drugs, and bronchodilators. Only RCTs were included. The outcomes of interest included the response rate, pulmonary function, and inflammation indicators.

### 2.2. Search Strategy

We searched PubMed, Embase, the Cochrane Library, SinoMed, CNKI, WanFang, VIP, and the Chinese Clinical Trial Registry to find relevant RCTs. The search date was from the inception of these databases up to November 1, 2021. The search strategy in PubMed, for example, was as follows: (“Pulmonary Disease, Chronic Obstructive” [mh] OR “chronic obstructive pulmonary disease”[tiab] OR “COPD”[tiab]) AND (“reduning” [tiab] OR “e du ning” [tiab]). All records yielded were imported into Endnote X9.3.3 for management.

### 2.3. Data Collection

Two team members independently screened the bibliography records according to the inclusion and exclusion criteria by reading the titles, abstracts, and full texts. They then extracted the data needed for this study, including authors, year of publication, sample size, course of disease, age, intervention and control measures, dose and course of treatment, and outcomes, and imported them into a pretested table. Any disagreements were resolved through consensus discussion.

### 2.4. Outcomes

The primary outcome was the response to treatment according to the “Guiding Principles for Clinical Research of New Chinese Medicines,” which is generally indicated by the symptoms of AECOPD (such as cough, wheezing, chest tightness, and dyspnoea) being relieved by more than 30%. Secondary outcomes included changes in pulmonary function (FEV_1_ and FEV_1_/FVC); changes in counts of T-lymphocytes (CD3, CD4, CD8, and CD4/CD8); changes in blood gas indicators (PH) such as hydrogen ion concentration, partial pressure of carbon dioxide (PaCO_2_), and partial pressure of oxygen (PaO_2_); changes in inflammation indicators such as C-reactive protein (CRP), and procalcitonin (PCT), and the incidence of adverse reactions.

### 2.5. Risk of Bias Assessment

The risk of bias of the RCTs was assessed using the Cochrane Risk of Bias Assessment tool. This tool includes seven items, namely, random sequence generation method, allocation hiding, blinding of patients and clinicians, blinding of outcome evaluators, data completeness, selective reporting, and other bias. Each item was rated as “low risk,” “high risk,” or “uncertain risk.”

### 2.6. Data Analysis

RevMan 5.4 software was used to perform the meta-analyses. For dichotomic data, relative risks (RRs) and 95% confidence intervals (CIs) were used as the effect size, with the Mantel–Haenszel method (M–H method) being used for incorporating data. For continuous data, the mean differences (MDs) were pooled by the inverse variance method, and the 95% CIs were given. All meta-analyses were based on a random effects model. Statistical heterogeneity between studies was assessed by the chi-squared test and I^2^ values, with significance levels of *p* < 0.10 and I^2^ > 50%, respectively. We performed subgroup analyses stratified by different courses of treatment (≤7 days versus >7 days) to explain the source of heterogeneity. Funnel plots and Egger's tests were used to detect publication bias for outcomes with at least 10 studies included.

## 3. Results

### 3.1. Results of the Search and Screening

**Figure 1:**
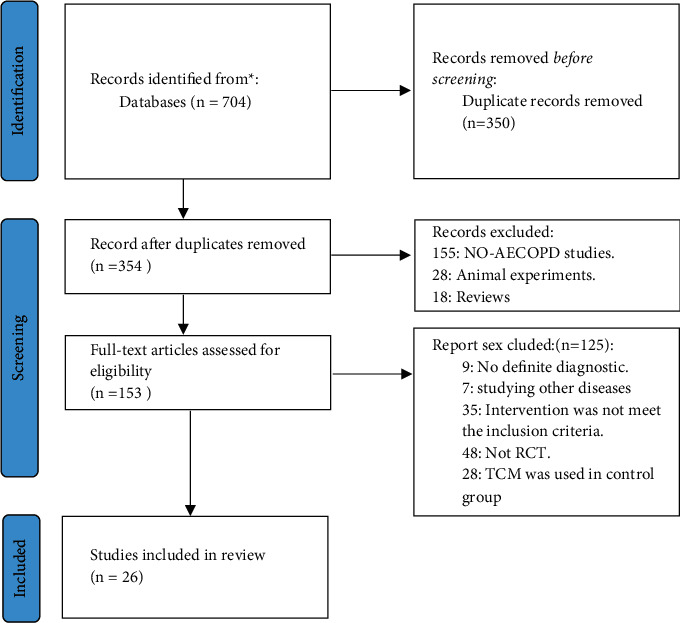
Process of literature screening.

A total of 704 records were identified, 350 of which remained after the exclusion of duplicates. After screening the titles, abstracts, and full texts, 26 RCTs [12–37] were included in our review.  Figure 1 shows the process of literature screening.

### 3.2. Descriptive Characteristics

The included RCTs enrolled a total of 2284 patients with AECOPD, with a sample size in individual RCTs ranging from 30 to 226. The average age was 55–73 years in the experimental (Re Du Ning) group and 54–76 years in the control group. Re Du Ning was administered through intravenous infusion in all RCTs, with a frequency of once a day and a dose of 20 ml/day. Anti-infective treatments were administered in all trials. The course of treatment was ≤7 days in 12 studies and >7 days in 16 studies. The detailed study characteristics are compiled in Table 1.

### 3.3. Results of Risk of Bias Assessment

**Figure 2:**
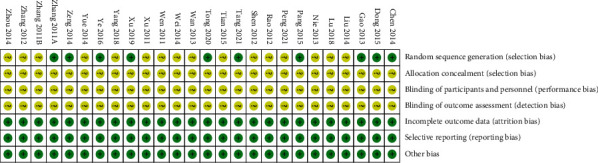
Results of the risk of bias assessment.

All 26 included studies claimed that allocation was random, but only 10 of them described the process of random number generation [13, 21, 22, 24–27, 30, 31]. None of the studies reported the application of allocation concealment or the status of blinding. There was no suspicion of selective reporting in any of the included studies, and no missing data were found. In general, all RCTs had an overall moderate to high risk of bias (Figure 2).

### 3.4. Response to Treatment

**Figure 3:**
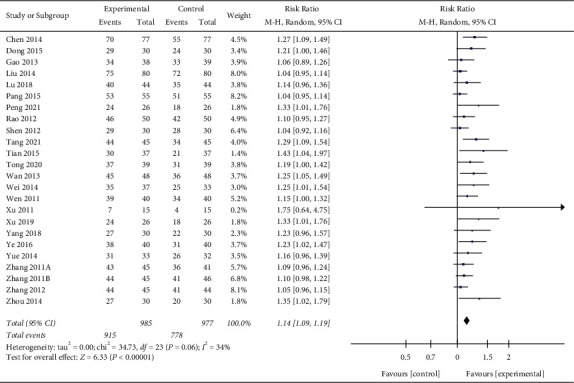
Forest plot of the meta-analysis of response to treatment.

Twenty-four studies [12–24,26–28, 30–37] reported the response rate to treatment, involving 1962 patients with AECOPD. The response rates were 92.8% and 79.6% in the experimental group and the control group, respectively. The pooled results demonstrated that the experimental group had a significantly better response rate than the control group (RR: 1.14%; 95% CI: (1.09, 1.19),*p* < 0.00001) (Figure 3), with low heterogeneity (I^2^ = 34%).

### 3.5. FEV_1_

**Figure 4:**
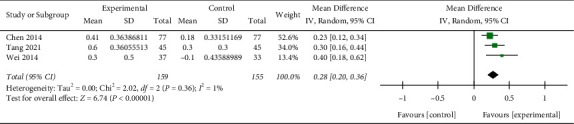
Forest plot of the meta-analysis of FEV1.

Three studies [17, 21, 27] (*n* = 314) reported on FEV_1_. Significantly more improvement in pulmonary function (FEV1) was obtained under with Re Du Ning injection (MD: 0.28 L; 95% CI: (0.20, 0.36),*p* < 0.00001) (Figure 4), with low heterogeneity (I^2^ = 1%).

### 3.6. FEV_1_/FVC%

**Figure 5:**
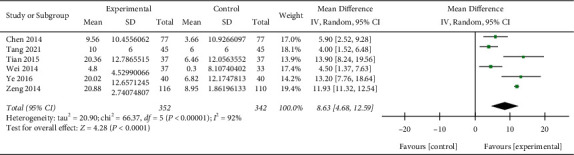
Forest plot of the meta-analysis of FEV1/FVC%.

Six studies [13, 17, 21, 25, 27, 36] (*n* = 694) reported on FEV1/FVC%. The treatment effect was more obvious in the Re Du Ning group than in the control group (MD: 8.63%; 95% CI: (4.68, 12.59);*p* < 0.0001) (Figure 5), with substantial heterogeneity (I^2^ = 92%).

### 3.7. pH, PaO2, and PaCO2

**Figure 6:**
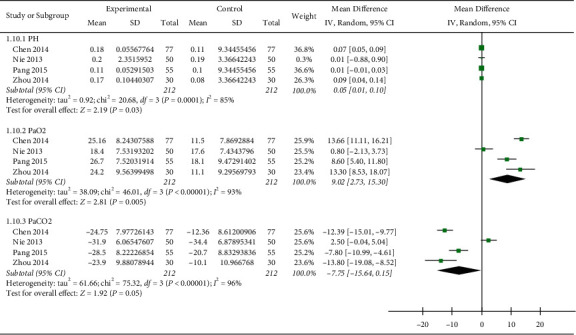
Forest plot of meta-analysis of PH, PaO_2_ and PaCO_2_.

Four studies [16, 21, 26, 29] (*n* = 414) reported data on indicators of blood gas analysis. As shown in Figure 6, the Re Du Ning injection significantly increased pH (MD: 0.05; 95% CI: (0.01, 0.10);*p*=0.03; I^2^ = 85%) and PaO2 (MD: 9.02 mmHg; 95% CI: (2.73, 15.3),*p*=0.005; I^2^ = 93%) when compared with the control treatment but did not significantly change PaCO_2_ (MD: −7.75 mmHg; 95% CI: (−15.64, 0.15);*p*=0.05; I^2^ = 96%) (Figure 6).

### 3.8. CRP

**Figure 7:**
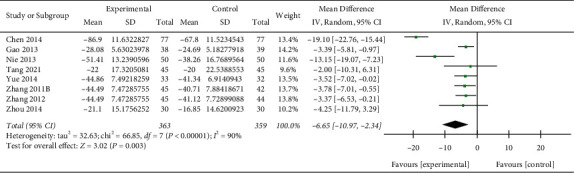
Forest plot of the meta-analysis of CRP.

Eight studies [14, 16, 19, 21, 24, 27, 29, 30] reported the level of CRP before and after treatment (*n* = 722). Meta-analysis suggested that the experimental group had a better change in CRP than the control group (MD: −6.65 mg/L; 95% CI: (−10.97, −2.34);*p*=0.003) (Figure 7), with substantial heterogeneity (I^2^ = 90%).

### 3.9. PCT

**Figure 8:**
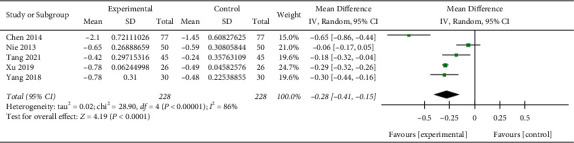
Forest plot of the meta-analysis of PCT.

Five studies [12, 21, 27, 29, 31] reported on PCT (*n* = 456). Meta-analysis suggested that the experimental group had a better change in PCT than the control group (MD: −0.28 *µ*g/L; 95% CI: (−0.41, −0.15);*p* < 0.00001) (Figure 8), with substantial heterogeneity (I^2^ = 86%).

### 3.10. T-Lymphocytes

**Figure 9:**
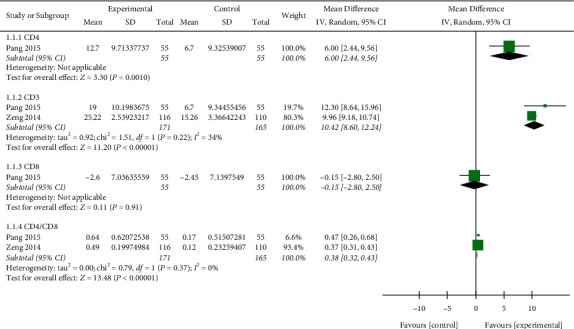
Forest plot of the meta-analysis of T-lymphocyte.

Only two trials [25, 26] (*n* = 336) reported on T-lymphocytes. As shown in Figure 9, compared with the control treatment, the Re Du Ning treatment significantly increased the CD4 count (MD: 6%; 95% CI: (2.44, 9.56);*p*=0.001), the CD3 count (MD: 10.42%, 95% CI: (8.6 to 12.24);*p* < 0.00001), and the ratio of CD4/CD8 (MD: 0.38%; 95% CI: (0.32 to 0.43);*p* < 0.00001) but did not significantly impact the CD8 count (MD: −0.15%; 95% CI: (−2.80 to 2.50);*p*=0.91).

### 3.11. Subgroup Analyses

According to the study settings in the PROSPERO protocol, we performed a subgroup analysis of the treatment response group, the CRP group, and treatment duration ≤7 and >7 days (details in Table 2).

### 3.12. Sensitivity Analysis

The sensitivity analyses changing the effect model to be fixed did not show any significant changes in the effect size for all outcomes.

### 3.13. Publication Bias

**Figure 10:**
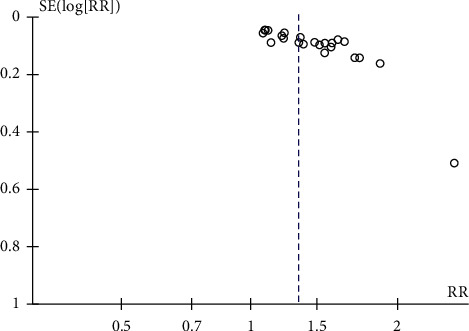
Funnel plot of response rate.

Publication bias was only available in the outcome of the response rate, which was included in at least ten studies. The funnel plot was asymmetrical (Figure 10), and the *p* value was <0.001 in the Egger test, confirming the existence of significant publication bias in this outcome.

### 3.14. Safety

Four studies reported adverse events after Re Du Ning treatment, including four cases of chest tightness, four cases of dizziness, eight cases of rash, and nine cases of gastrointestinal reactions. No serious adverse events were reported.

## 4. Discussion

With the ageing of the population, environmental pollution, and urbanization, COPD as a global challenge is becoming increasingly difficult. Traditional Chinese medicine injections composed of herbal extracts provide a new approach for the treatment of COPD. A total of 26 studies were included in this study, and the results of the meta-analysis showed that Re Du Ning injection had a significant adjuvant effect on the improvement of clinical symptoms and pulmonary function in patients with acute exacerbations of COPD, as well as reducing inflammatory factors and correcting the acid-base imbalance in the body.

AECOPD is mostly caused by respiratory infections. CRP and PCT have attracted attention as clinically sensitive indicators of inflammation and bacterial infection. According to the results of the meta-analysis, the Re Du Ning injection reduced the levels of CRP and PCT. Pharmacological analyses have shown that *Gardenia*, honeysuckle, and *Artemisia annua*, the components of Re Du Ning injection, have anti-inflammatory, antibacterial, and antiviral effects, and their active chemicals include iridoids, lignans, coumarins, sesquiterpenoids, flavonoids, caffeoylquinic acids, and phenolic acids [38, 39]. *Artemisia annua* is widely known as the source of artemisinin, discovered by academician Youyou Tu, and in addition to killing malaria parasites, its antibacterial activity against a variety of Gram-negative bacteria/positive bacteria is outstanding [40]. Iridoids and flavonoids have antitussive, expectorant, antiasthmatic, and antibacterial activities. Geniposide (the main iridoid in Gardenia) and chlorogenic acid in honeysuckle can inhibit acute and chronic inflammatory disease-related macrophage responses, reduce the release of inflammatory factors, lower C-reactive protein, and suppress infection at the same time [41]. Pharmacological experiments also showed that geniposide can protect pulmonary artery smooth muscle from lipopolysaccharide damage by affecting the tlr-4/myd88 signalling pathway, improve pulmonary function, and avoid the formation of pulmonary hypertension [42]. These mechanisms confirm the authenticity of the results of this study on FEV1 and FEV_1_/FVC, suggesting that Re Du Ning may be a new approach for improving exercise tolerance and delaying pulmonary function decline in patients with COPD.

Acute exacerbations of COPD often lead to carbon dioxide retention and hypoxemia. Severe cases can be complicated by respiratory acidosis, resulting in respiratory depression, respiratory failure, pulmonary encephalopathy, and even death. The improvement in pulmonary function after the Re Du Ning treatment was also reflected in the results of blood gas analyses—the findings of the meta-analysis indicated that the hypoxemia and acid-base imbalance in the experimental group were improved, and the acidosis of the body was corrected. The results of PaCO_2_ were not statistically significant, which may be impacted by Nie et al.'s study [29] with a treatment course of only 3 days, which was much shorter than that of the other 3 studies. There is no evidence on the effect of Re Du Ning injection on direct airway expansion, and therefore its effects on pulmonary function and blood gas indicators may be associated with its efficacy on respiratory tract inflammation and infection.

Re Du Ning injection seemed to be well tolerated in patients with COPD, as indicated by only mild adverse reactions reported in four out of 26 RCTs. However, there is no lack of adverse reactions caused by Re Du Ning injection in previous reports. A survey on adverse reactions of Re Du Ning injection reported 1452 adverse reactions, of which 952 (65.9%) were reported by minors and were mostly immediate, occurred within 60 minutes of the first administration, and commonly manifested as local skin rashes; however, some adverse reactions may also be severe in a small proportion of patients [43]. The analysis by high-performance liquid chromatography on ultrafiltration of Re Du Ning injection suggested that allergic reactions may be associated with Tween-80 (made by the polymerization of dehydrated sorbitol monooleate and ethylene oxide, used as a solvent or emulsifier for injection and oral liquid) [44]. According to the “Basic Principles of Clinical Use of TCM Injections” issued by the Chinese government, the adverse reactions of this injection can be prevented to a certain extent by strengthening the safety monitoring of the first administration, avoiding combined administration with other injections, and controlling the drip rate [45].

There are some limitations in this study. First, the included RCTs did not mention the implementation process of blinding, which may cause performance bias. Second, the criteria of efficacy assessments were not entirely consistent across the RCTs, and the accuracy of effect estimates may thus be impacted. Third, the heterogeneity in the meta-analyses was high in some outcomes, and the subgroup analysis stratified by the length of treatment did not explain the source of heterogeneity.

## 5. Conclusion

The current RCT evidence shows that Re Du Ning injection is an effective and safe complementary therapy based on routine treatments for AECOPD, as indicated by improvements in the response rate, pulmonary function, acid-base imbalance, inflammatory indicators, and mild adverse reactions. The quality of evidence was impacted by the risk of bias and heterogeneity in the RCTs of the meta-analysis. More well-designed RCTs are warranted to further validate the efficacy and safety of Re Du Ning injection on AECOPD.

## Figures and Tables

**Table 1:** Studies characteristics.

Author	Sample size (EG/CG)	Mean age (EG/CG)	Experimental intervention	Cointerventions	Course of treatment (days)	Outcomes
Zeng et al. [25]	116/110	—	RDN 20 ml ivgtt qd	Antibiotics; bronchodilator; PRM; AD	10	C, D
Chen et al. [21]	77/77	64.7/65.8	RDN 20 ml ivgtt qd	Antibiotics; PRM; AD	7	A, B, C, E, F, G, H, I
Chen et al. [23]	30/30	—	RDN 20 ml ivgtt qd	Antibiotics; bronchodilator; AD; oxygen therapy	10	A
Dong et al. [22]	30/30	62.3/61.3	RDN 20 ml ivgtt qd	Antibiotics; bronchodilator; PRM	7	A
Tian 2015 [36]	37/37	59.7/60.8	RDN 20 ml ivgtt qd	Antibiotics; PRM; AD	10	A, C
Zhang et al. [24]	45/41	55/57	RDN 20 ml ivgtt qd	Antibiotics; PRM; AD	3	A
Liu et al. [34]	80/80	68.2/66.8	RDN 20 ml ivgtt qd	Antibiotics; PRM; AD	14	A
Yao [33]	50/50	62.1/62.4	RDN 20 ml ivgtt qd	Antibiotics; bronchodilator PRM; AD; oxygen therapy	7	A
Weng et al. [32]	40/40	57/58	RDN 20 ml ivgtt qd	Antibiotics; bronchodilator	5	A
Xu et al. [28]	15/15	—	RDN 20 ml ivgtt qd	Antibiotics; bronchodilator; PRM; AD; oxygen therapy	7	A
Gao et al. [30]	38/39	66/63	RDN 20 ml ivgtt qd	Antibiotics; AD; oxygen therapy	5	A, H
Nie et al. [29]	50/50	72.4/70.2	RDN 20 ml ivgtt qd	Antibiotics; oxygen therapy	3	E, F, G, H, I
Tang [27]	45/45	63/63	RDN 20 ml ivgtt qd	Antibiotics; PRM; oxygen therapy	14	A, B, C, H, I
Pang et al. [26]	55/55	55/54	RDN 20 ml ivgtt qd	Antibiotics; PRM; AD; oxygen therapy	14	A, D, E, F, G
Lu [20]	44/44	65.9/67.1	RDN 20 ml ivgtt qd	Antibiotics; bronchodilator; PRM	7	A
Wan [18]	48/48	58.9/59.5	RDN 20 ml ivgtt qd	Antibiotics; bronchodilator; PRM	7	A
Wei [17]	37/33	—	RDN 20 ml ivgtt qd	Antibiotics; bronchodilator; PRM	10	A, B, C
Zhou 2014 [16]	30/30	72.6/73.1	RDN 20 ml ivgtt qd	Antibiotics; bronchodilator; PRM	7	A, E, F, G, H
Zhang [14]	45/44	66.2/65.7	RDN 20 ml ivgtt qd	Antibiotics; bronchodilator; PRM; AD; oxygen therapy	10	A, H
Ye et al. [13]	40/40	62.8/62.4	RDN 20 ml ivgtt qd	Antibiotics; bronchodilator; PRM	28	A, C
Yang et al. [12]	30/30	64.5/64.2	RDN 20 ml ivgtt qd	Antibiotics; bronchodilator; PRM; AD; oxygen therapy	10	A, I
Zhang et al. [35]	45/42	66.2/65.7	RDN 20 ml ivgtt qd	Antibiotics; PRM; AD	10	A, H
Yue [19]	33/32	73/70	RDN 20 ml ivgtt qd	Antibiotics; AD	14	A, H
Peng et al. [15]	26/26	65.0/62.9	RDN 20 ml ivgtt qd	Antibiotics; PRM; AD	10	A
Tong [37]	39/39	64.2/63.8	RDN 20 ml ivgtt qd	Antibiotics; AD	10	A
Xu et al. [31]	26/26	62.7/61.4	RDN 20 ml ivgtt qd	Antibiotics; PRM	10	A, I

EG: experimental group; CG: control group; PRM: phlegm resolving medicine; AD: antiasthmatic drugs; RDN: Re Du Ning injection; ivgtt: intravenous drip; qd: once a day; A: response rate; B: FEV_1_; C: FEV_1_/FVC; D: T-lymphocyte E: PH; F: PaO_2_; G: PaCO_2_; H: C-reactive protein; I: procalcitonin.

**Table 2:** Results of subgroup analysis.

Outcome	Course of treatment (days)	No. of studies	Effects within subgroups (95% CI)	Heterogeneity within subgroups	Interaction *p* value
Response rate	≤7	9	RR 1.15 (1.09, 1.22)	*p*=0.53; I^2^ = 0%	*p*=0.74
	>7	14	RR 1.14 (1.07, 1.20)	*p*=0.02; I^2^ = 36%	

C-reactive protein (mg/L)	≤7	4	MD −10.06 (−19.05, −1.07)	*p* < 0.00001; I^2^ = 94%	*p*=0.16
	>7	4	MD −3.48 (−5.33, −1.63)	*p*=0.98; I^2^ = 0%	

## Data Availability

The data supporting this research article are available from the corresponding author on reasonable request.

## Abbreviations

RDN: Re Du Ning injection
COPD: Chronic obstructive pulmonary disease
AECOPD: Acute exacerbation of chronic obstructive pulmonary disease
PRM: Phlegm resolving medicine
AD: Antiasthmatic drugs
Ivgtt: Intravenous drip
qd: Once a day
RR: Relative risk
MD: Mean difference
CI: Confidence interval.
